# The JMU-SalVac-System: A Novel, Versatile Approach to Oral Live Vaccine Development

**DOI:** 10.3390/vaccines12060687

**Published:** 2024-06-20

**Authors:** Andreas Iwanowitsch, Joachim Diessner, Birgit Bergmann, Thomas Rudel

**Affiliations:** 1 Chair of Microbiology, University of Würzburg, 97074 Würzburg, Germany; andreas.iwanowitsch@uni-wuerzburg.de; 2 Department of Obstetrics and Gynecology, University Hospital Würzburg, 97080 Würzburg, Germany; diessner_j@ukw.de

**Keywords:** balanced-lethal system, vaccine development, Ty21a, CTB

## Abstract

*Salmonella enterica* Serovar Typhi Ty21a (Ty21a) is the only licensed oral vaccine against typhoid fever. Due to its excellent safety profile, it has been used as a promising vector strain for the expression of heterologous antigens for mucosal immunization. As the efficacy of any bacterial live vector vaccine correlates with its ability to express and present sufficient antigen, the genes for antigen expression are traditionally located on plasmids with antibiotic resistance genes for stabilization. However, for use in humans, antibiotic selection of plasmids is not applicable, leading to segregational loss of the antigen-producing plasmid. Therefore, we developed an oral Ty21a-based vaccine platform technology, the JMU-SalVac-system (Julius-Maximilians-Universität Würzburg) in which the antigen delivery plasmids (pSalVac-plasmid-series) are stabilized by a Δ*tyrS*/*tyrS*^+^-based balanced-lethal system (BLS). The system is made up of the chromosomal knockout of the essential tyrosyl-tRNA-synthetase gene (*tyrS*) and the in trans complementation of *tyrS* on the pSalVac-plasmid. Further novel functional features of the pSalVac-plasmids are the presence of two different expression cassettes for the expression of protein antigens. In this study, we present the construction of vaccine strains with BLS plasmids for antigen expression. The expression of cytosolic and secreted mRFP and cholera toxin subunit B (CTB) proteins as model antigens is used to demonstrate the versatility of the approach. As proof of concept, we show the induction of previously described in vivo inducible promoters cloned into pSalVac-plasmids during infection of primary macrophages and demonstrate the expression of model vaccine antigens in these relevant human target cells. Therefore, antigen delivery strains developed with the JMU-SalVac technology are promising, safe and stable vaccine strains to be used against mucosal infections in humans.

## 1. Introduction

An efficient and cost-effective approach to combat mucosal infections is to stimulate the mucosal immune system via live attenuated bacterial vaccines administrated via mucosal routes. One of the key issues in developing a bacterial vaccine system is to find an optimal balance between attenuation of the vaccine strain to ensure safety and sufficiently high expression of vaccine antigens to trigger an immune response. Excessive attenuation often leads to reduced immunogenicity due to reduced fitness and an impaired ability to colonize and persist in lymphoid tissues (reviewed in [1]). In addition, high antigen expression can also have a negative effect on immunogenicity by impairing metabolism and reducing fitness [2]. To develop an effective, safe and stable antigen expression system using live *Salmonella* bacteria as a vehicle, an optimal ratio between antigen expression and fitness of the vaccine strain must therefore be achieved.

The expression of heterologous antigens in live bacterial vector strains by the incorporation of stable plasmids is a flexible and easy to realize method for the production of new vaccines, especially in comparison to the genomic integration of expression cassettes. The expression of antigens via high-copy number plasmids may allow a higher production of antigens than expression via the genome but may have the disadvantage that it affects the fitness of the vehicle strain. There are different ways to circumvent negative effects of high antigen expression on the fitness of the vaccine strain, e.g., the use of plasmids with lower copy number or the use of promoters that can be induced in vivo (regulated delayed protein synthesis). In addition, suitable sub-cellular targeting can optimize the production of the antigen and the fitness of the vaccine strain and significantly improve the strength and type of immune response (reviewed in [3,4]).

It has been shown that secretion of the vaccine antigens, in contrast to cytosolic expression, increases the immunogenicity of the antigens and additionally reduces metabolic burden on live oral vaccines [4,5]. The *hlyA* secretion system of *Escherichia coli* has already been used successfully for secretion of antigens [6,7]. The *hly* operon consists of four essential components, the transcription enhancer sequence *hlyR*, the HlyA acylating enzyme HlyC and the inner membrane complex HlyB-HlyD [8]. HlyB transports antigens which have been fused to the *hlyA*-signal across the inner membrane, while HlyD interacts with TolC in the outer membrane for secretion into the lumen [8]. Actively exported antigens become accessible to the host immune system without the bacteria being degraded [8,9,10].

Antigenicity of the heterologous protein is another key factor for successful vaccination with live vaccines. Uptake and presentation by antigen-presenting cells (APCs) is a decisive step, which may be enhanced by peptide fusions targeting APCs, such as the non-toxic subunit of the cholera toxin CtxB [11]. CTB pentamers bind to monosialotetrahexosylganglioside (GM1) present on gut epithelial cells and APCs, even when antigens are fused to it [12]. Immunization strategies in which CTB is administered as adjuvant either as CTB antigen fusion or as recombinant CTB show enhanced immune responses [13,14]. CTB adjuvants have been classified as safe by the regulatory authorities, as demonstrated by their use in one of the three approved vaccine formulations against *Vibrio cholerae*, Dukoral^®^.

When using plasmids for antigen expression in vaccine strains, one challenge is to ensure vector stability. Although antibiotics are useful and effective in providing plasmid stability under selective conditions in vitro, their use in vivo is usually not applicable and moreover raises safety concerns in cases where the goal is to generate a product for administration to humans. Even the use of antibiotics in fermentation cultures bears the risk that the final product is contaminated with residual antibiotics that in turn could potentially cause an allergic reaction in sensitive individuals [15]. Furthermore, regulatory agencies recommend avoiding antibiotic resistance genes in plasmids due to the risk of antibiotic resistance being transferred to other organisms in the environment, especially pathogens [16,17].

The stabilization of plasmids in live vaccines must therefore be achieved by other means, in particular by balanced-lethal host-vector systems (BLS), where, for example, a gene coding for an essential protein is deleted from the host chromosome and complemented on the plasmid. One of the first and most prominent BLSs is the well-studied *asd* BLS. The protein encoded by the aspartate-β-semialdehyde dehydrogenase (asd) gene is an enzyme involved in the biosynthesis of diaminopimelic acid (DAP) from aspartate. Deletion of asd (Dasd) makes the bacteria auxotrophic for DAP resulting in lysis when the bacteria grow in absence of DAP. By integrating a functionally active asd expression cassette into the plasmid, the chromosomal deletion can be complemented, and the plasmid can be stably maintained in the carrier in the absence of DAP. Since DAP hardly occurs in animal or human organism, the loss of the plasmid leads to lysis of the Dasd bacteria, as they can no longer synthesize peptidoglycan [18]. Although promising results were obtained in a mouse models [19,20,21], clinical trials with a S. Typhi Ty2-derived ∆asd vaccine strains were disappointing [22,23]. It was speculated by Galen et al. that the catalytic activity of Asd could be rescued by a single or few copies of *asd*, likely leading to suboptimal maintenance of antigen-expressing plasmids [24]. To circumvent this fundamental problem, the authors developed an alternative BLS based on the non-catalytic single-stranded DNA-binding protein (SSB), whose crucial function is to prevent unstable ssDNA intermediates [24]

In addition to proteins essential for the biosynthesis of peptidoglycan like Asd, the thymidylate synthase ThyA, an enzyme involved in synthesis of nucleic acid building blocks was employed for the design of an auxotrophy-based BLS [25].

Based on the considerations above, we developed an antigen expression platform technology (JMU-SalVac-system) in which the antigen delivery plasmids (pSalVac-plasmid-series) are stabilized by a Δ*tyrS*/*tyrS*^+^-based balanced-lethal system in Ty21a. The system consists of the chromosomal knockout of the essential gene *tyrS*, which encodes for the tyrosyl-tRNA-synthetase, and the in trans complementation of this gene on pSalVac-plasmids. The pSalVac-plasmids enable the simultaneous expression of two heterologous proteins. In this study, we demonstrate the stability of the plasmid, the expression of mRFP and the secretion of the mucosal adjuvant CTB from the vaccine strain Ty21a. The model antigens mRFP and CTB are expressed in infected human primary macrophages under the control of in vivo inducible promoters, demonstrating the application of the JMU-SalVac-system in these central immune cells. In summary, we have developed a system in which antigens can be stably, safely and efficiently expressed, indicating that bacterial strains developed with our system have the potential to be used as safe and stable vaccine strains against mucosal infections suitable for use in humans.

## 2. Methods and Materials

### 2.1. Rational Design

To ensure 100% maintenance of Ty21a cells with the desired antigen delivery plasmids, we decided to develop an antibiotic resistance gene-free balanced-lethal system that is not based on auxotrophy, but on complementing the chromosomal deletion of an essential gene on pSalVac plasmids. As an essential gene we chose *tyS*for several specific reasons. The *tyrS*gene is a single copy gene in the *Salmonella* genome with a short coding sequence of 1275 bp. Although aminoacyl-tRNA synthetases are evolutionarily very old and highly conserved, some members, such as TyrS, exhibit species-specific recognition. Crucially, prokaryotic and human cytoplasmic tyrosyl-tRNA synthetases cannot acylate their respective counterpart’s tRNA [26], which excludes the possibility of cross-complementation and ensures the dependence of the bacterial carrier on the BLS plasmid. We decided to choose the *tyrS* gene from *E. coli* K-12 instead of the native Salmonella *tyrS*gene as a recombinant complementing gene on the extrachromosomal vector to reduce the potential risk of unwanted integration during the chromosomal recombination step. The *tyrS*gene in *E. coli* is located within the same flanking genes as its counterpart in the Salmonella genome and shows 88% nucleotide identities with the respective Salmonella gene, whereas both gene products have 96% amino acid identity. Furthermore, the *E. coli tyrS*gene locus has been well studied with regard to promoter structure, regulation of expression and termination of the *tyrS*gene [27,28]. Preliminary studies of *tyrS*-6xHis-expression in *E. coli* under control of its own wildtype promoter showed high levels of synthesis of the TyrS-6xHis protein and a reduction in growth rate (see Supplementary Figure S6). To avoid toxic effects of *tyrS*-overexpression, we decided to reduce *tyrS*-expression by placing the gene under the control of a putative weaker promoter. It is known that in *E. coli*, the LacI repressor which regulates expression of the lactose metabolic genes by binding to the lacO operator sequence [29] is constitutively synthesized at a very low level of about 5 to 10 copies per cell [30,31]. Thus, to reduce the expression on each single plasmid and therefore favor the regulation of expression towards a higher plasmid copy number, the *tyrS*-6xHis-coding sequence was cloned under the control of a *lacI*-derived promoter (PlacI-like) and integrated into pSalVac-plasmids.

### 2.2. Standard Microbial and Molecular Methods Used for Generation of JMU-SalVac Strains

If not noted otherwise, *E. coli* DH5α (Invitrogen, Darmstadt, Germany) was utilized for subcloning, plasmid amplification and maintenance. *S. enterica* serovar Typhi strain Ty21a and its Δ*tyr*S derivatives were used as the basis for the generation of antigen expression strains. Unless otherwise stated, bacterial strains were grown aerobically in LB broth Lennox vegetal (Carl Roth, Karlsruhe, Germany) at 37 °C with rigorous shaking (180–200 rpm), or on LB-Agar (Lennox) vegetal (Roth). Antibiotic selection was performed as required with ampicillin (Sigma-Aldrich), kanamycin (Sigma-Aldrich, Darmstadt, Germany) and chloramphenicol (Sigma-Aldrich) at final concentrations of 100, 25 and 20 µg/mL, respectively. All bacterial strains and plasmids used in this study are listed in Table 1. Primers are listed in Supplementary Table S1. Standard molecular methods were performed following published protocols [32]. PCR-products and digests were purified either with QIAquick PCR Purification Kit (Qiagen) or the QIAquick Gel Extraction Kit (Qiagen, Hilden, Germany) following the manufacturer’s recommendations. Plasmids were purified with QIAprep Spin Miniprep Kit (Qiagen) and QIAGEN Plasmid Midi Kit (Qiagen) following the manufacturer’s instructions.

### 2.3. Construction of the BLS-Recipient Strain S. enterica Typhi Ty21a ∆tyrS(tyrS CmR)^+^

For the construction of the chromosomal *tyrS*-knockout we modified the method of “one-step inactivation of chromosomal genes using PCR products” which was described by Datsenko and Wanner, 2000 [33]. As *tyrS* is an essential gene, a knockout without genetic compensation would be lethal. Therefore, we first inserted a functional TyrS-expression cassette into the PCR-template-plasmid pKD3. For excision, the tyrS expression cassette and the chloramphenicol resistance gene are flanked by two FRT sites. Thus, during recombination, the chromosomal *tyrS* is not only replaced by a fragment encoding for the antibiotic resistance, but also by a gene encoding *E. coli tyrS*. In brief, we introduced the *tyrS* gene upstream of CmR and amplified the resulting FRT-*tyrS*-CmR-FRT-region with primers featuring homologous overhangs to the genomic *tyrS*. After electroporation of the PCR product into *S. enterica* Typhi Ty21a harboring the helper plasmid pKD46, a temperature-sensitive Red helper plasmid which carries the Red recombination system with the phage ג Red recombinase under control of an arabinose-inducible promoter [35], one Cm-resistant transformant was selected and correct integration of the FRT-*tyrS-*CmR-FRT-Fragment replacing the *tyrS*-locus was confirmed via PCR and sequencing. After removal of the pKD46 plasmid by incubation at 37 °C, the selected clone was made electrocompetent and transformed with the helper plasmid pCP20. The resulting strain (*S. enterica* Typhi Ty21a ∆*tyrS* (*tyrS* CmR)^+^ pCP20) represents the balanced-lethal-system-recipient strain of our JMU-SalVac-system: TyrS complementing pSalVac-plasmids which are intended to be BLS-stabilized are transformed into Ty21a ∆*tyrS* (*tyrS* CmR)^+^ pCP20for generation of the final antibiotic resistance gene free JMU-SalVac antigen delivery strains.

### 2.4. Design and Construction of the pSalVac Antigen Delivery Plasmids

**Figure 1:**
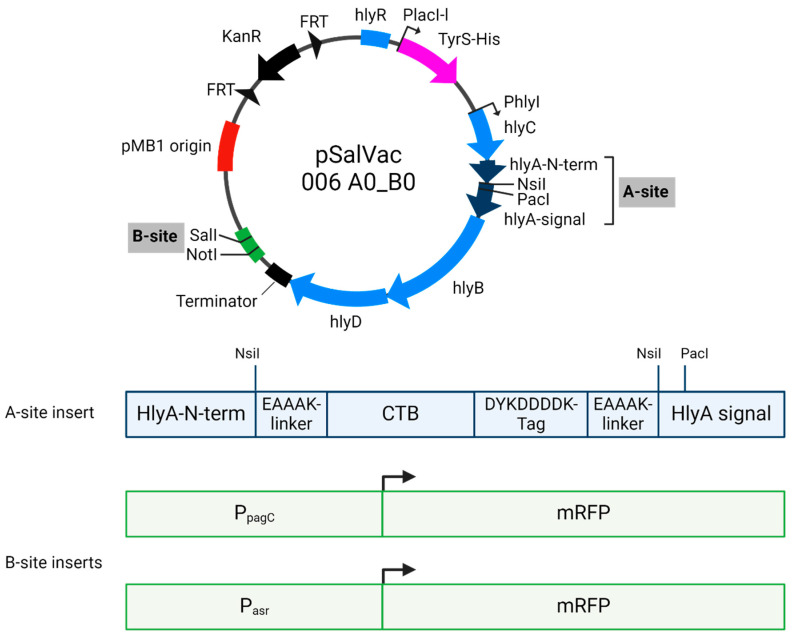
Genetic map of pSalVac base vector (**top**) and the inserts (**bottom**). The base vector contains the *hlyA* secretion cluster including a cloning site (A-site) for the insertion of secreted antigens such as CTB-FLAG. Another independent cloning site (B-site) is located downstream of the *hlyA* cluster. A kanamycin resistance cassette flanked by FRT-sites can be excised via Flippase expression and TyrS is expressed from a lacI-derivative promoter for balance-lethal stabilization in the Δ*tyrS* host. Figure created with BioRender.com.

The basic pSalVac antigen delivery plasmid pSalVac 006 A0_B0 KanR (see Figure 1) forms the basis of the various antigen delivery plasmids of the pSalVac Ax_By KanR-series (x and y represent placeholders for different antigen-expressing cassettes) used in this study. It is derived from the first generation pSalVac 001 A0_B0 KanR (Patent No. WO 2022/034221 A1) plasmid which has a pMB1 origin of replication (about 20 copies per cell [39]). For selection in vitro, it has a kanamycin resistance expression cassette (KanR) that is flanked by two sites of flippase recognition targets (FRT-sites [10]). This allows the excision by the site-specific enzyme FLP recombinase, which acts on the direct repeats of the FRT-sites [33]. Functional features of the pSalVac Ax_By KanR- series are two independent expression cassettes for the different adjuvant and/or antigen-fusion proteins. The first expression cassette, named A-site, consists of the transcription enhancer sequence *hlyR*, the structural genes *hlyC*, *hlyB* and *hlyD* and two short residual sequences of the *hlyA* gene separated by a single NsiI-restriction site (integration site of in silico designed coding sequence of planned fusion protein). A terminator sequence was introduced after the *hly*D coding sequence using complementary primers. As the integration site for the complementing *tyrS* expression cassette on the antigen delivery plasmids, we selected a region within the *hly* operon between the enhancing sequence *hlyR* and *hly*C (Figure 1). This region contains an IS2 sequence which has been shown to have no effect on HlyA secretion [40]. The activity of the *hlyR* enhancer is dependent on the correct spacing from the *hlyC* orf, however, which is why we chose to replace IS2 with the TyrS cassette. This ensures the correct spacing between *hlyR* and *hlyC*, while eliminating the unnecessary IS2 transposon element. A multiple cloning site for the second expression cassette (B-site), was integrated into the unique SalI site of pSalVac 001 A0_B0 KanR. For the generation of the different plasmids of the pSalVac Ax_By series, the NsiI-fragments (x) were cloned into the A-(NsiI)-expression site, whereas the B-site-fragments (y) were cloned into the B-(MCS)-expression site of the pSalVac 006 A0_B0 KanR vector. For this study, we used the inactive cholera enterotoxin B-subunit CTB as model antigen. Rigid EAAAK-linker were used to connect the CTB and the adjacent sequence to facilitate domain formation and improve adjuvant efficacy [41]. Java Codon Adaptation Tool (JCAT) (http://www.jcat.de/ accessed on 24 June 2020) [42] was used for codon optimization of the coding sequences to *S. enterica* Typhi (strain ATCC 700931/Ty2). The optimized NsiI-Linker-ctxB-Linker-FlagTag-Linker-NsiI sequence was synthesized (Thermo Fisher, Darmstadt, Germany) and introduced into the pSalVac 006 A0_B0 KanR plasmid via In-Fusion cloning (Takara Biotech, Saint-Germain-en-Laye, France) resulting in pSalVac A_ctxB__B0 KanR. For an additional control strain, one FRT site flanking the KanR was deleted from this plasmid via In-Fusion to prevent KanR excision, resulting in the BLS-ActxB_B0 KanR variant after stabilization. For intracellular expression analysis, previously described in vivo inducible promoters P_asr_ (acid induced [43]) and P_pagC_ (low Mg^2+^ [44]) were cloned upstream of mRFP using In-Fusion cloning (Takara Biotech) according to the manufacturer’s protocol (see Figure 1). Promoter sequences were amplified from the Ty21a genome and ribosome binding sides were optimized with according primers using the UTR designer tool (https://sbi.postech.ac.kr/utr_designer/reverse/ accessed on 9 November 2022 [45]). The dual reporter system was amplified from pFCcGi [34] and cloned into pSalVac 006 A0_B0 KanR using In-Fusion cloning. Sequences of all plasmids used in this study were routinely verified by whole-plasmid sequencing (Microsynth, Balgach, Switzerland).

### 2.5. Generation of BLS-Stabilized Vaccine Strains

**Figure 2:**
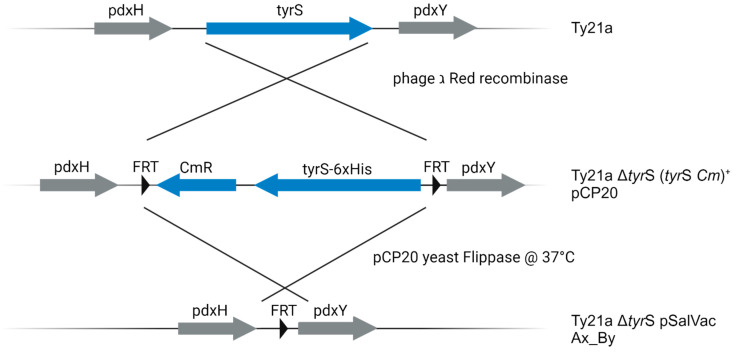
Schematic representation of the genomic *tyrS* locus of Ty21a during generation of BLS-stabilized JMU-SalVac strains. First, the *tyrS* locus of the licensed Ty21a strain was replaced with a *tyrS*-CmR cassette flanked by FRT sites. After transformation with one of the TyrS complementing pSalVac-plasmids, the *tyrS* cassette is deleted by expression of yeast flippase encoded on pCP20, leaving behind a short nucleotide sequence with one FRT site. Figure created with BioRender.com.

The presence of FRT sites flanking the chromosomally integrated (*tyrS* CmR)^+^ cassette and the kanamycin resistance gene on the *tyrS*-complementing pSalVac plasmids enables the simultaneous deletion of those loci by flippase, yielding a BLS-stabilized strain which has no residual antibiotic resistance genes (see Figure 2). In contrast to the method described by Datsenko and Wanner [33], not only the FRT-flanking fragment in the chromosome but also the FRT-flanking kanamycin resistance gene cassette in the plasmid had to be eliminated. To assure elimination of all FRT flanked sequences, we established a modified protocol for the elimination step. In brief, electrocompetent cells of BLS-R were transformed with one of the pSalVac antigen delivery plasmids. After 1 h incubation at 30 °C in LB broth without antibiotics, kanamycin/ampicillin/chloramphenicol triple resistant transformants were selected at 30 °C on LB agar plates containing 25 µg/mL kanamycin and 100 µg/mL ampicillin as described [33]. Ampicillin is required for selection of pCP20. KanR, AmpR, and CmR-positive clones were cultivated in LB-Medium containing 100 µg/mL ampicillin and 25 µg/mL kanamycin at 30 °C overnight. The next day, the culture was diluted 1:1000 in LB-Medium containing only 100 µg/mL ampicillin and cultivated for 1 h at 37 °C and subsequently for 1 h at 30 °C. These two steps were repeated at least four times. Incubation at 37 °C leads to the expression of yeast flippase from the pCP20 plasmid [35] to simultaneously eliminate the kanamycin-(plasmid) and *tyrS* CmR (genome) cassettes, while recovery at 30 °C serves to replenish copies of pCP20, which does not replicate at 37 °C. This approach is an adaptation of the original method of Datsenko and Wanner (2000) [33] which is necessary to quantitatively remove all FRT-flanked sequences (from the chromosome and the plasmid). After the temperature shifts, the cultures were selected on LB-Amp at 30 °C [33] and single colonies were screened for kanamycin sensitivity. Kanamycin-sensitive clones were then inoculated into LB-Medium without antibiotics and shaken at 37 °C overnight to eliminate pCP20. Single colonies were tested for sensitivity to ampicillin, kanamycin, and chloramphenicol. Additionally, the correct excision of the genomic *tyrS* locus as well as the kanamycin locus on the plasmid were confirmed by colony PCR.

### 2.6. Plasmid Stability Assay

Plasmid maintenance in vitro was determined by serial passage of bacteria cultures without any selective pressure. In brief, overnight cultures of tested strains were diluted 1:40 in LB-Medium without (only BLS-ActxB_B0) or supplemented with 25 µg/mL kanamycin until mid-logarithmic phase to generate generation 0. Subsequently, these starter cultures were diluted 1:1000 into fresh LB-Medium and cultured to stationary phase. In the same way, bacterial cultures were passaged up to 6 times. Each day, serial dilutions of the strains harboring plasmids with kanamycin resistance gene were plated on TS-agar plates without antibiotic selection and incubated at 37 °C for 18–24 h to obtain single colonies. At least 100 colonies per day and strain-harboring plasmids with kanamycin resistance gene were selected randomly and grown on fresh TS agar plates containing 25 µg/mL kanamycin and on TS agar without antibiotics. In case of the investigated antibiotic resistance-free BLS-stabilized vaccine strain (BLS-ActxB_B0), cultures of day 0 and 6 were serially diluted and plated on TS agar plates. After incubation overnight at 37 °C, at least 100 colonies were picked on TS agar. The presence of the BLS-stabilized plasmid (ΔKanR) in the investigated strain was monitored by PCR amplification assays using plasmid-specific primers.

### 2.7. Bacterial Growth Assay

Overnight cultures were diluted to an OD_600_ of 0.1 and the OD_600_ was measured at 37 °C under shaking in triplicate over 22 h using a TECAN MPlex microplate reader and iControl 2.0 software (Tecan Trading AG, Männedorf, Switzerland). Pathlength correction was not applied.

### 2.8. Western Blot Analysis

Whole cell lysates (WCL) of bacterial cultures grown to OD_600_ 1.5 (mid-logarithmic phase) were prepared by pelleting at 4000 x rcf. Resulting supernatant fractions were precipitated with 20% final concentration of ice-cold TCA according to standard protocols. Samples were analyzed on an SDS-PAGE under reducing conditions and transferred to PVDF. After blocking in 5% skimmed milk powder for 1 h, the primary antibodies (anti-DYKDDDDK Tag, Cell Signalling Cat. No. 14793S or Anti-His Tag, GenScript Cat. No. 25B6E11) were incubated overnight at 4 °C. HRP-coupled secondary antibodies (Biozol, Hamburg, Germany) were applied 1:20,000 for 1 h at room temperature and developed with Femto ECL (Thermo Fisher) using an INTAS Professional imaging system (INTAS Science Imaging, Göttingen, Germany).

### 2.9. Differentiation of Human-Monocyte-Derived Macrophages (hMDMs)

Primary human macrophages were derived from peripheral blood mononuclear cells (PBMCs) isolated from leukoreduction system cones using the SepMate™-50 system (StemCell Technologies, #85450) and Ficoll-Paque (GE Healthcare, #17144003) gradient according to manufacturer’s instructions. Monocytes were purified from the PBMC fraction using the EasySep CD14+ system (StemCell Technologies, #17858) according to manufacturer’s instructions and seeded in 12-well cell culture plates, at a density of 2 × 10^5^ cells/well, in RPMI1640 (Thermo Fisher Scientific, #72400054) supplemented with 10% (*v*/*v*) heat-inactivated FBS and 50 ng/mL recombinant human macrophage colony-stimulating factor (M-CSF) (StemCell Technologies, #78057). Macrophages were allowed to differentiate for 7 days and used for experiments on day 8. For infections, 2 × 10^5^ hMDMs were infected with freshly cultured bacteria at MOI10. After 2 h infection, the cells were washed with PBS and extracellular bacteria were killed with 15 µg/mL gentamycin (Thermo Fisher) for 30 min before the medium was changed to 1 µg/mL gentamycin for the remining total infection duration as indicated.

### 2.10. Infection of RAW 246.7 Cells

RAW 246.7 cells (=ATCC TIB71) were routinely cultured in RPMI1640 supplemented with 1X GlutaMaXX and 10% heat-inactivated FCS (Thermo Fisher). For infections, 1 × 10^5^ RAW 246.7 cells were seeded the day before in a 12-well plate and infected with freshly cultured bacteria at MOI25. After 2 h infection, the cells were washed with PBS and extracellular bacteria were killed with 15 µg/mL gentamycin (Thermo Fisher) for 30 min before the medium was changed to 1 µg/mL gentamycin for the remaining total infection duration as indicated.

### 2.11. Flow Cytometry Analysis

After infection, RAW 246.7 or hMDMs cells were washed three times with PBS and a live/dead stain was performed with FVS660 (Becton Dickinson, Temse, Belgium) for 15 min at 4 °C. Afterwards, cells were fixed for 15 min with ROTI^®^Histofix (Roth) at room temperature. Cells were permeabilized in 0.1% Triton-X for 15 min, blocked in 1% BSA and 22.25 mg/mL glycine for 1 h and primary antibody (BD Difco™ *Salmonella* O Antisera) was applied in 1:500 dilution for 1 h. Secondary antibody (Donkey anti-Rabbit IgG (H+L) Highly Cross-Adsorbed Secondary Antibody, Alexa Fluor™ 488, Invitrogen) was applied for 1 h at room temperature. Cells were scraped and analyzed with an Attune NxT flow cytometer. Gates were set based on negative controls (non-infected for Ty21a+ve and empty vector for mRFP+ve, see Supplementary Figure S5). Statistical analysis was performed with GraphPad Prism software version 10.0.

### 2.12. Immunofluorescence

Raw 246.7 cells or hMDMs seeded onto coverslips were washed three times after infection and fixed for 15 min with ROTI^®^Histofix (Roth) at room temperature. Cells were permeabilized in 0.1% Triton-X for 15 min, blocked in 1% BSA and 22.25 mg/mL glycine for 1 h and primary antibody (BD Difco™ *Salmonella* O Antisera) was applied in 1:1000 dilution for 1 h. Secondary antibody (Donkey anti-Rabbit IgG (H+L) Highly Cross-Adsorbed Secondary Antibody, Alexa Fluor™ 488, Invitrogen) was applied for 1 h at room temperature. The slides were mounted with ProLong™ Glass mounting medium with NucBlue DNA stain.

## 3. Results

**Figure 3:**
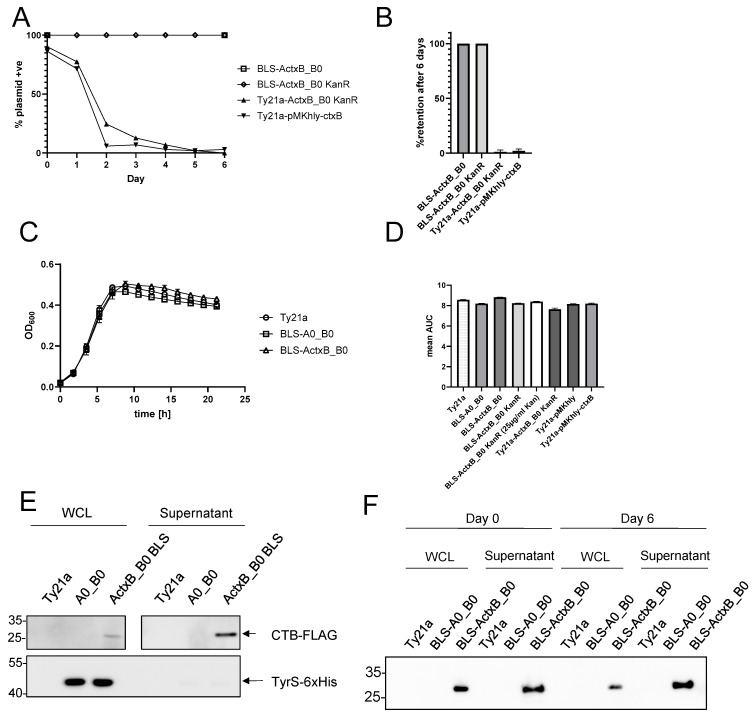
Maintenance of BLS plasmids and effect on growth behavior and antigen expression. (**A**) In vitro plasmid stability of non-stabilized (Ty21a ActxB_B0 KanR, Ty21a pMKhly-ctxB) and BLS-stabilized (BLS ActxB_B0, BLS-ActxB_B0 KanR) strains. For details, see Section 2. (**B**) endpoint plasmid retention of three independent replicates of the plasmid stability assay. (**C**) Growth curves of WT Ty21a and BLS-stabilized vaccine strains (*N* = 3). (**D**) Area under the curve analysis (AUC) of growth curves of different *S.* Typhi/plasmid combinations. Data were obtained from three independent replicates (error bars represent standard deviation, *N* = 3) (**E**) Western blot analysis of BLS-ActxB_B0, whole cell lysate (**left**) and supernatants (**right**). Approx. 7.5 × 10^5^ bacteria per well (for whole cell lysate, WCL) and 5 µg total protein per well (supernatant) were analyzed by SDS-PAGE (10% gel) before transfer to PVDF. Detection of CTB-FLAG was performed with anti-FLAG primary antibody and TyrS-6xHis with anti-6xHis primary antibody. (**F**) Western blot analysis before (Day 0) and after (Day 6) plasmid stability assay.

We developed a Ty21a-based vaccine platform technology (JMU-SalVac-system) in which the antigen delivery plasmids (pSalVac-plasmid-series) are stabilized by a Δ*tyrS*/*ty*r*S*^+^ based balanced-lethal system (BLS). In order to test the feasibility of the JMU-SalVac-system, we first constructed several BLS-stabilized vaccine strains (see Table 1). These include strains with the CDS of mature CTB as model antigen in A-site (see Figure 1) for secretion via the *hlyA*-secretion system (abbreviation BLS-ActxB_B0), the mRFP-gene integrated into the B-site (see Figure 1) under control of the in vivo inducible promoter *asr* (BLS-A0_B_Pasr_RFP), the mRFP-gene integrated into the B-site and under control of in vivo inducible promoter *pag*C (BLS-A0_B_PpacC_mRFP) and a control strain with empty A- and B-sites (BLS-A0_B0). Further control strains were a Ty21a wildtype vaccine strain transformed with plasmid pSalVac A_ctxB__B0 KanR (Ty21a-ActxB_B0 KanR) and a BLS-stabilized strain with a kanamycin resistance gene retained on the pSalVac plasmid (BLS-ActxB_B0 KanR). The latter strain serves as a reporter strain for the plasmid retention status in colonies of the BLS strain. The kanamycin resistance gene of BLS-A_ctxB__B0 KanR is not needed for the retention of the plasmid but facilitates readout during the plasmid stability assay. To test stability of the Δ*tyrS*/*tyrS*^+^ BLS, both BLS-stabilized strains BLS-A_ctxB__B0 and BLS- A_ctxB__B0 KanR as well as the non-stabilized kanamycin-resistant strain Ty21a-A_ctxB__B0 KanR were tested for plasmid stability by serial passages of cultures at 37 °C in LB-medium for 6 days in the absence of antibiotic selection. For comparison, strain Ty21a harboring the conventional antigen expression plasmid pMKhly-CtxB [10] was also included. Plasmid maintenance in strains harboring plasmids with KanR genes was determined by examining resistance to kanamycin daily. The ratio of colonies growing on TS agar with kanamycin compared to TS agar without kanamycin was calculated. For the BLS-stabilized strain BLS-ActxB_B0, which does not carry an antibiotic resistance gene, a plasmid-specific PCR analysis was performed with at least 100 colonies from TS agar plated from serial dilutions of the starter culture (generation 0) and after 6 days of serial passaging. Plasmids without BLS stabilization showed low stability, as less than 80% of the picked colonies were able to grow on kanamycin containing TS-agar after just one day of serial passage (see Figure 3A). After 6 days, this fraction of kanamycin resistant colonies dropped to less than 3%. In contrast, both BLS-stabilized pSalVac plasmids showed 100% retention after 6 days of serial passage (for absolute numbers see Supplementary Table S2). No change in nucleotide sequence of the CTB-expressing A-site of BLS-ActxB_B0 was observed in 10 sequenced clones after 6 days of passage, and an mRFP-expressing BLS strain (BLS-ActxB_BDR) showed mRFP expression in 100% of tested colonies after the same 6-day passage duration.

**Figure 4:**
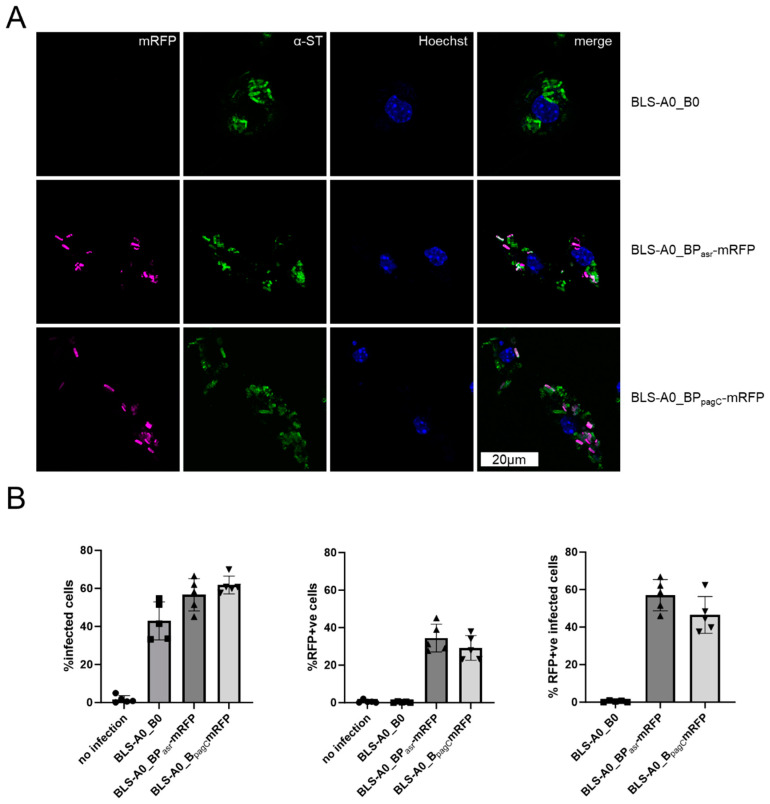
In vivo inducible promoters in BLS-stabilized plasmids are active in Ty21a-infected RAW246.7 macrophages. Immunofluorescence images (**A**) and flow cytometry analysis (**B**) of RAW246.7 cells infected with Ty21a strains with in vivo inducible promoters driving mRFP expression. Empty vector (BLS-A0_B0) was used as negative control (e.v.) for 2h. Extracellular bacteria were killed with 15 µg/mL gentamycin for 30 min and further 2.5 h (**B**) or 3.5 h (**A**) with 1 µg/mL gentamycin. Cells were stained with fixable viability stain (B only), fixed, permeabilized and stained with anti-Typhi primary antibody and Alexa488 secondary before analysis. (**B**) All live cells were gated for infection ((**first**) panel), mRFP expression ((**middle**) panel), or sub-gated for infected cells with mRFP signal ((**right**) panel). Data were obtained from five independent replicates (error bars represent standard deviation, *N* = 5).

Effective immunization with live vector vaccines depends on high and sustained antigen expression, for which plasmid stability is crucial. To simultaneously ensure the fitness of the vaccine strains, the pSalVac vector system enables the efficient secretion of soluble antigens via the *hlyA* secretion system, limiting the toxicity of difficult-to-express antigens. Antigens destined for secretion are cloned in the designated A-site to create a fusion protein with the *hlyA* N-terminus and the *hlyA*-signal fused N- and C-terminally, respectively. This enables expression via the constitutively active *hlyA* promoter and secretion of the protein as shown here for CTB-FLAG (Figure 3E). The CTB-FLAG signal in the supernatant does not originate from autolysis of the vaccine strains, as virtually none of the cytosolically expressed TyrS-His was detected in the supernatant. Expression of CTB did not significantly alter the growth behavior of the carrier strain, as shown by growth curve analysis (Figure 3C) and the corresponding area under the curve analysis of the growth curves (Figure 3B). Furthermore, expression of CTB-FLAG did not change after 6 days of serial passage (Figure 3F). In addition to the A-site, the so-called B-site, which is located downstream of the *hlyA* secretion apparatus genes, can be used for expression. We illustrate this in this study with in vivo inducible promoters such as the acid shock response promoter (P_asr_) and SPI-II promoter P_pagC_, which enable the regulated delayed antigen expression of otherwise toxic proteins with minimal loss of fitness for the carrier [2,46]. P_asr_- and P_pagC_-mRFP promoter fusions were cloned into the B-site of pSalVac and transformed into a BLS-stabilized vaccine strain. These strains were used to infect the macrophage cell line RAW246.7 (Figure 4). Microscopical and flow cytometry analysis of infected cells shows that mRFP is expressed intracellularly in up to ~30% of all macrophages, with P_asr_-mRFP in above 50% of infected macrophages (see Figure 4B right panel).

**Figure 5:**
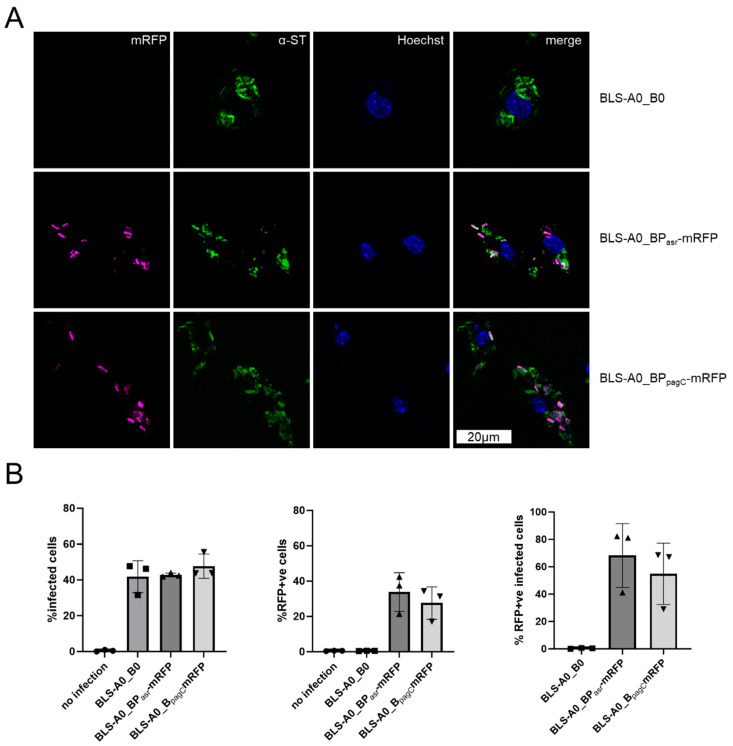
In vivo inducible promoters in BLS-stabilized plasmids are active in Ty21a-infected hMDMs. Immunofluorescence images (**A**) and flow cytometry analysis (**B**) of human-monocyte-derived macrophages (hMDMs) infected at MOI10 with BLS strains with in vivo inducible promoters driving mRFP expression. BLS-A0_B0 was used as negative control (e.v.). hMDMs were infected with Ty21a strains expressing mRFP under the control of P_asr_ (A0_BP_asr_mRFP) or P_pagC_ (A0_BP_pacC_mRFP) for 2 h. Extracellular bacteria were killed with 15 µg/mL gentamycin for 30 min and further 2.5 h with 1 µg/mL gentamycin. Cells were stained with fixable viability stain ((**B**) only), fixed, permeabilized and stained with anti-Typhi primary antibody and Alexa488 secondary before analysis. (**B**) All live cells were gated for infection ((**first**) panel), mRFP expression ((**middle**) panel), or sub-gated for infected cells with mRFP signal ((**right**) panel). Data were obtained from three independent replicates (error bars represent standard deviation, *N* = 3).

To demonstrate the expression of model antigens by P_asr_ and P_pagC_ in a model relevant to vaccination of humans, human-monocyte-derived macrophages (hMDMs) were infected with the BLS-stabilized Ty21a reporter strains. Both promoters (Figure 5) were induced in infected primary hMDMs, indicating that intracellular vaccine strains encounter an environment permissive for promoter induction.

**Figure 6:**
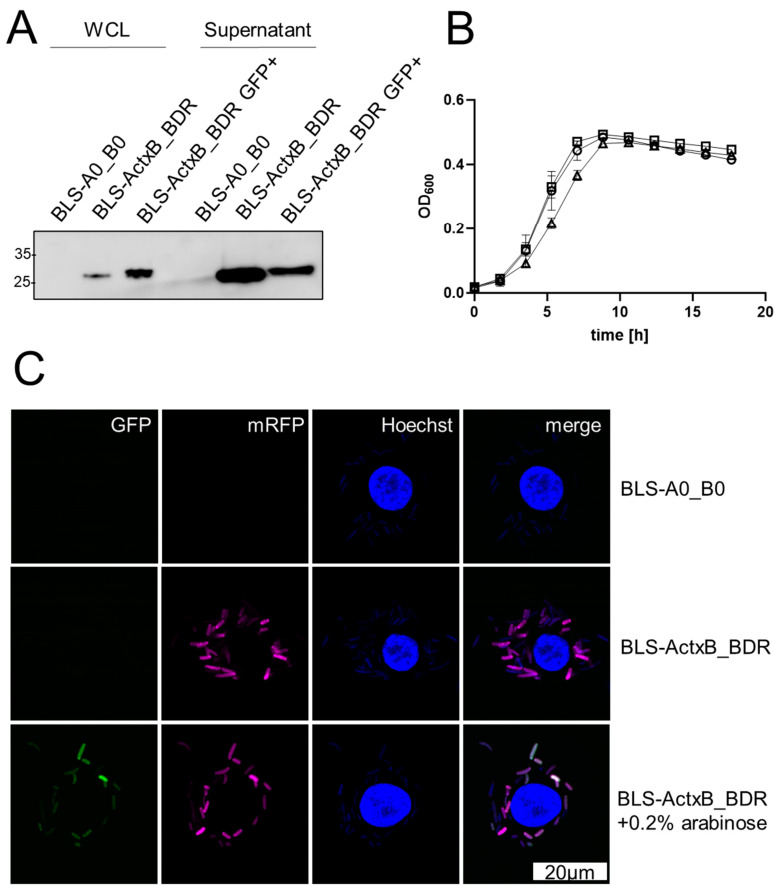
Simultaneous expression of two antigens and intracellular replication of BLS strains in hMDMs. BLS strain constitutively expressing CTB-FLAG and the dual reporter system mRFP and arabinose inducible GFP (BLS-ActxB_BDR) visualized by (**A**) western blot analysis of whole cell lysate and TCA precipitated supernatant, (**B**) growth curve analysis and (**C**) hMDM infection. (**A**) Approx. 7.5 × 10^5^ bacteria per well (for whole cell lysate, WCL) and 5µg precipitated supernatant were analyzed on a 12% SDS-PAGE before transfer to PVDF. Detection of CTB-FLAG was performed with anti-FLAG primary antibody. GFP+ denotes induction with 0.2% arabinose. (**B**) Growth curves of WT Ty21a (circles) and BLS-stabilized vaccine strains BLS-A0_B0 (boxes) and BLS-ActxB_BDR (triangles) (*N* = 3) (**C**) hMDMs were infected for 2 h with arabinose induced *S.* Typhi BLS-ActxB_BDR. Extracellular bacteria were killed with 15 µg/mL gentamycin for 30 min and further 2.5 h with 1 µg/mL gentamycin and mounted with ProLong mounting medium containing NucBlue.

Administration of a combination of multiple antigens may prevent immune escape of different serovars, but the simultaneous expression of different antigens from the same vaccine host may increase metabolic burden and reduce fitness. Using both expression cassettes for expression of secretory and cytosolic antigens (CTB-FLAG and mRFP), we tested invasion and intracellular antigen expression in hMDMs. To monitor intracellular replication of the strains, a fluorescence reporter cassette on pFCcGi with constitutive mRFP and arabinose-inducible GFP expression was cloned into the B-site of ActxB_B0. Since GFP expression is only induced before the infection, the signal is diluted upon proliferation of *Salmonella* after infection and only the RFP fluorescence is retained. Non-proliferating bacteria retain both fluorescence signals. The BLS-stabilized strain expressed CTB-FLAG with and without induction of GFP via arabinose (Figure 6A) but showed a slight reduction in replication as shown by growth curve measurements (Figure 6B). Nevertheless, BLS-ActxB_BDR proliferated in the hMDM infection model, as revealed by varying GFP signal strength in mRFP-positive bacteria in the hMDMs (Figure 6C). While live cell imaging also showed intracellular replication in hMDM on the single cell level, bacterial fluorescence decreased across the whole field of view during the measured time course (see Supplementary Movie S1 and Figure S9). These data indicate that the BLS-stabilized carrier strain expressing heterologous proteins in A- and B-site still has sufficient fitness to infect and survive in the hMDM model for a substantial period.

## 4. Discussion

We developed a plasmid system for antigen expression and delivery for *Salmonella enterica* serovar Typhi strain Ty21a (Ty21a), the approved attenuated bacterial vaccine against typhoid fever. This vaccine strain has been administered to more than 200 million recipients worldwide over 25 years and has never become virulent again [47], which emphasizes its excellent safety profile. The effect of pre-existing immunity to Salmonella carrier strains on the response to heterologous antigens has been investigated in several studies with mixed results, some showing an enhanced immune response [48,49,50] and others a reduction [51,52] (reviewed in [53]). Most relevant to the present work, studies on Ty21a showed no effect [54] or even an enhancement of the immune response against the heterologous antigen in carrier-primed human vaccinees [49]. The authors speculate that the choice of antigen is an important factor in determining the outcome of vaccination in such individuals [49]. Although Ty21a has been established as a versatile oral vaccine vector for expression of various foreign antigens [55], there is currently no stable, non-auxotrophic, non-antibiotic resistance plasmid system available for antigen expression in Ty21a. The JMU-SalVac-system described in this study closes this important gap.

As the use of antibiotics or other external treatments for plasmid stabilization in vaccinees is not possible, other selection mechanisms had to be developed to ensure retention of the expression plasmids (for recent review about Salmonella-based carrier vaccine strains, see [22]. This has previously been achieved in *Salmonella-*based vaccine strains, e.g., by the knockout of the aspartate β-semialdehyde dehydrogenase (Asd) and in trans complementation on the expression plasmid [56]. Stabilization after chromosomal deletion of *asd* results in DAP auxotrophy. However, plasmids stabilized by in trans complementation of auxotrophies may be lost if, e.g., auxotrophies in essential metabolic pathways are complemented by environmental metabolites.

We used a different strategy here by deleting the essential *tyrS* gene in the genome of Ty21a and complementation of *tyrS* on a low to medium copy vector, resulting in a non-antibiotic and non-auxotrophic stabilization. Our data show that the Δ*tyrS*/*ty*r*S*^+^ based JMU-SalVac-system provides stability of the plasmid without further selection, whereas non-stabilized plasmids were rapidly lost under the same conditions. Since the *tyrS* gene is essential and functional complementation by environmental metabolites is excluded [26,57], the stability of the JMU-SalVac system should also be given in complex in vivo situations. Furthermore, in trans complementation of *tyrS* had no negative impact on the fitness of the vaccine strain, determined by growth and infection behavior. This is of crucial importance as Ty21a is already strongly attenuated by mutations in several virulence genes, which makes it a good candidate for safe mass vaccination from the outset. In addition, the expression of model antigens remained stable during the tested 6-day passage of the strains, which is important for a long-lasting production of antigens during the vaccination phase (see Figure 3). Our system overcomes the challenges of previous approaches to non-auxotrophic stabilization. SSB-based stabilization has shown expression instability after several days of passage, which is not seen with the TyrS-based BLS, as evidenced by sustained resistance to kanamycin in the BLS-ActxB_B0 KanR strain, mRFP expression in all colonies of BLS-ActxB_BDR and unchanged expression of CTB-FLAG after 6 days of passage (Figure 3F) [24]. Higher copy number plasmids, which may be advantageous for high antigen expression, were also no problem for plasmid stability with the TyrS-stabilized vectors as opposed to stabilization with SSB [24]. In addition, this is the first such system described for the Ty21a vaccine strain.

Important functional features of the pSalVac plasmids are two different sites for expression and delivery of antigen proteins. The first expression cassette (A-site) includes the *hly* operon and two short residual sequences of the *hlyA* N-terminus and the C-terminal signal sequence (hlyAs) for recognition and secretion via the hemolysin secretion system [9,40]. One advantage of using the *hlyA* system for secretion of antigens into the extracellular space is that a relatively short secretion signal of the *hlyA* toxin has to be fused to the antigen for efficient secretion. In this study we use CTB as model antigen and show efficient secretion into the culture supernatant. CTB was selected here since it has very strong adjuvant activity, particularly for the induction of mucosal immunity [58,59]. Protein antigens fused to CTB are stabilized and CTB directs the antigen fusion protein to its receptor ganglioside GM1 for uptake by antigen-presenting cells.

The second expression cassette (B-site) is located outside the *hly* gene cluster and is variable with respect to promoter, signal peptide and thus also the expression strength and the subcellular localization of the fusion protein. The simultaneous expression of different antigens, which was shown here with CTB-FLAG (A-site) and mRFP (B-site), can be advantageous to avoid immune escape of pathogens. The two different expression cassettes thus offer a high degree of flexibility, e.g., with regard to the expression and subcellular localization of vaccine antigens, and represent an alternative to published dual plasmid systems [20].

Oral vaccination with encapsulated lyophilized Ty21a ensures the successful passage of the bacteria through the gastric acid barrier [60]. In the small intestine, the bacteria invade the mucosa and translocate to the intestinal lymphoid follicles and the draining mesenteric lymph nodes, where they interact with mononuclear phagocytic cells of the lymphoid follicles. We therefore tested infection of RAW246.7, and particularly human-monocyte-derived macrophages (hMDMs) as relevant target cells for Ty21a BLS strains. In contrast to the infection experiments with P_asr_-mRFP reporter fusions performed by others [43], in which Ty21a was unable to induce active infection of THP-1 derived macrophages, the strains constructed here with a similar reporter fusion infected and in some cases even multiplied in macrophages as was measured with a dual reporter system and live cell imaging (see Figure 6 and Supplementary Movie S1) [34]. As expected, bacterial fluorescence decreased during the time course of live cell imaging however (Supplementary Figure S9), which indicates that the majority of internalized Ty21a is degraded by the hMDM cells. The eventual degradation of the vaccine host is important for the delivery of non-secreted antigens as was modelled here with mRFP. The use of in vivo inducible antigen expression under the control of the acid-inducible P_asr_ and the Mg^2+^-sensing P_pagC_ revealed strong expression in the majority of cells. Infected cells without mRFP expression likely had not yet acidified (in the case of the asr construct) or depleted the Mg^2+^ concentration enough for the reporter to be visible in flow cytometry at the time of measurement. Such in vivo-inducible promoters can facilitate the immunization with difficult to express antigens, limit the metabolic burden of antigen expression, and can have favorable effects on vaccination outcome, possibly due to increased fitness of the strongly attenuated carrier strain [22].

The next step is to validate the developed expression system in a suitable in vivo model. However, even positive immunization results obtained in mice or other animals are not indicative of an effective vaccine, as Ty21a cannot infect animals orally. Since *S.* Typhi is an exclusively human pathogen, clinical studies are the most important next step for the validation of antigen expression systems [61]. This will show if the non-auxotrophic approach here is an improvement over previously demonstrated BLSs that failed to induce protection with Ty21a in the clinical setting [62,63].

In summary, we have constructed and tested a novel antibiotic resistance gene-free, non-auxotrophic plasmid stabilization system for Ty21a-based live vaccines in which antigens can be stably, safely, and efficiently expressed. Bacterial strains developed with our system have the potential to be used in humans as safe and stable vaccines against mucosal infections.

## 5. Patents

A patent covering the construction and use of pSalVac vectors has been filed (WO 2022/034221 A1).

## Figures and Tables

**Table 1:** Bacterial strains and plasmids.

Plasmids/Bacteria	Relevant Characteristics	Expression Cassettes	Antibioitic Resistance Genes	Source or Reference
Plasmids				
pKD46	Helper plasmid, Red recombinase, g, b and exo from ParaB promoter		AmpR	[33]
pKD3	*bla FRT cat FRT PS1 PS2 oriR6K*		CmR	[33]
pKD3-SpeI	*bla FRT BcuI-site cat FRT PS1 PS2 oriR6K*		CmR	This study
pKD3*-*SpeI *tyrS* HisTag-s	*bla FRT P_WT_ tyrSx6His*, *cat FRT PS1 PS2 oriR6K*		CmR	This study
pMKhly1	*FRT KanR FRT*, *hlyR*, *hlyC*, *hlyAs*, *hlyB*, *hlyD*		KanR	[10]
pMKhly-CtxB	derivate of pMKhly1	*ctxB-hlyAs-fusion*	KanR	[10]
pSalVac 001 A0_B0 KanR	*FRT KanR FRT*, pMB1-origin, ΔIS2:P_lacI-like_*tyrS*-His_Tag_	A-Site*: hlyR*, *hlyC*, *hlyA-secretion signal*, *hlyB*, *hlyD* B-site: SalI site for integration	KanR	Patent No. WO 2022/034221 A1
pSalVac 006 A0_B0 KanR	derivate of pSalVac 001 A0_B0 KanR: deletion of residual Promotor region of AmpR, terminator downstream of *hlyD*, *MCS in B-site*		KanR	This study
pSalVac ActxB_B0 KanR	Derivate of pSalVac 006 A0_B0 KanR	A: *ctx*B*-hlyA*s fusion	KanR	This study
pSalVac ActxB_B0 KanR ∆FRT	Derivate of pSalVac ActxB_B0 KanR, downstream FRT site deleted	A: *ctx*B*-hlyA*s fusion	KanR	This study
pSalVac ActxB_B0 KanR P_wt_-TyrS	Derivate of pSalVac ActxB_B0 KanR, wildtype TyrS promotor	A: *ctx*B*-hlyA*s fusion	KanR	
pSalVac A0_BP_asr_-mRFP KanR	Derivate of pSalVac 006 A0_B0 KanR	B: P_asr_-mRFP (low pH)	KanR	This study
pSalVac A0_BP_pagC_-mRFP KanR	Derivate of pSalVac 006 A0_B0 KanR	B: PagC-mRFP (low Mg^2+^)	KanR	This study
pFCcGi	Inducible expression of GFP and constitutive expression of mCherry		AmpR	[34]
pSalVac ActxB_BDR KanR	Derivate of pSalVac ActxB_B0 KanR,	A: *ctx*B*-hlyA*s fusion B: mRFP and GFP expression cassettes (dual reporter, DR) of pFCcGi	KanR	This study
pCP20	helper plasmid, *CmR bla cat cI857 lPR flp pSC101 oriTS*		AmpR, CmR	[35]
*E. coli*				
DH5α	F^−^, ø80d*lacZ* M15, (*lacZYA-argF*)U169 *deoR*, *recA*1, *endA*1, *hsdR*17(rk^−^, mk^+^), *phoA*, *supE*44, λ^−^, *thi*-1, *gyrA*96, *relA*1		-	Invitrogen
CC118 (λpir)	*Δ(ara-leu)*, *araD*, *ΔlacX74*, *galE*, *galK*, *phoA20*, *thi-1*, *rpsE*, *rpoB*, *argE(Am)*, *recA*, *λpir phage lysogen*		-	[36]
*S.* *typhi*				
Ty21a	*S.* Typhi Ty2, *galE*, *rpoS*, *viaB*		-	[37,38]
Ty21a Δ*tyrS* (*tyr*S *Cm*R)^+^	Ty21a derivate, *tyrS* gene replacement by a (FRT *tyrS* *Cm*R FRT)^+^-knock-in-Fragment		CmR	This study
Ty21a Δ*tyrS*	Complemented by one of the BLS-stabilized pSalVac plasmids described above		-	This study
Strain name	Host/plasmid combination			
BLS-A0_B0	Ty21a Δ*tyrS* pSalVac 006 A0_B0 ΔKanR		-	This study
BLS-ActxB_B0	Ty21a Δ*tyrS* pSalVac ActxB_B0 ΔKanR	A: *ctx*B*-hlyA*s fusion	-	This study
BLS-ActxB_B0 KanR	Ty21a Δ*tyrS* pSalVac ActxB_B0 KanR ∆FRT	A: *ctx*B*-hlyA*s fusion	KanR	This study
BLS-ActxB_B0 P_wt_-TyrS	Ty21a Δ*tyrS* pSalVac ActxB_B0 P_wt_-TyrS	A: *ctx*B*-hlyA*s fusion	-	This study
BLS-A0_BP_asr_-mRFP	Ty21a Δ*tyrS* pSalVac A0_BP_asr_-mRFP ΔKanR	B: P_asr_-mRFP (low pH)	-	This study
BLS-A0_BP_pagC-_mRFP	Ty21a Δ*tyrS* pSalVac A0_BP_pagC_-mRFP ΔKanR	B: P_pagC_-mRFP (low Mg^2+^)	-	This study
BLS-ActxB_BDR	Ty21a Δ*tyrS* pSalVac ActxB_BDR ΔKanR	A: *ctx*B*-hlyA*s fusion	-	This study
Ty21a-ActxB_B0 KanR	Ty21a pSalVac ActxB_B0 KanR		KanR	This study
Ty21a-pMKhly-ctxB	Ty21a pMKhly1-CtxB		KanR	[10]
Ty21a-pMKhly	Ty21a pMKhly1		KanR	[10]

## Data Availability

The data presented in this study are available in this article and Supplementary Materials.

## Supplementary Materials

The following supporting information can be downloaded at: https://www.mdpi.com/article/10.3390/vaccines12060687/s1, Figure S1: Uncropped western blot picture of Figure 3. Expression of CTB-FLAG in supernatant fractions (SN); Figure S2: Uncropped western blot picture of Figure 3. Expression of CTB-FLAG in whole cell lysate (WCL); Figure S3: Uncropped western blot picture of Figure 3. Detection of TyrS-His in whole cell lysate (WCL) and supernatant fractions (SN); Figure S4: Uncropped western blot picture of Figure 6; Figure S5: Gating strategy for flow cytometry experiments; Figure S6: Growth curve analysis of BLS-ActxB_B0 Pwt-TyrS; Figure S7: Uncropped western blot picture of Figure 3F; Figure S8: Plasmid maps of key vectors used in this study; Figure S9: Relative mRFP fluorescence intensity during live cell imaging infection with BLS-ActxB_B0 in hMDM; Table S1: Primers; Table S2: Raw data for Figure 3; Movie S1: live cell imaging.
