# Supplementary material for: The JMU-SalVac-System: A Novel, Versatile Approach to Oral Live Vaccine Development
Source: Vaccines (Basel). 2024 Jun 20;12(6):687. doi: 10.3390/vaccines12060687 (PMC11209359; doi:10.3390/vaccines12060687)
Supplement: Supplementary file 1 [file vaccines-12-00687-s001.zip › Supplementary Figures.pdf]

Supplementary information

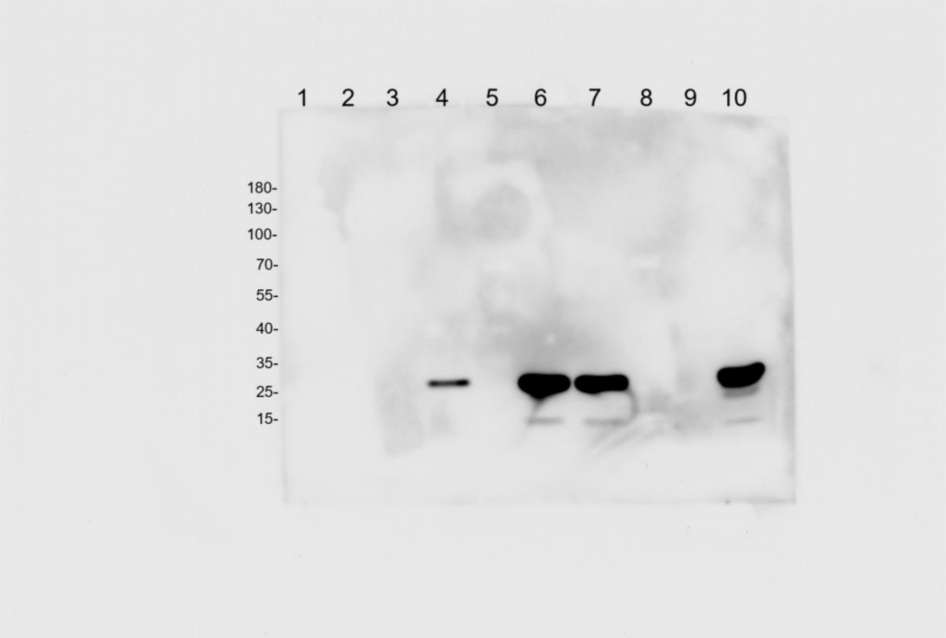

Supplementary Figure S1. Uncropped western blot picture of Figure 3. Expression of CTB-FLAG in supernatant fractions (SN) Lanes 1: PageRuler Prestained Protein ladder (Thermo Fisher), 2: Supernatant (SN) Ty21a 3: SN BLS-A0_B0, 4: SN BLS-ActxB_B0, 5: PageRuler Prestained Protein ladder (Thermo Fisher), 6-10: unrelated experiments.

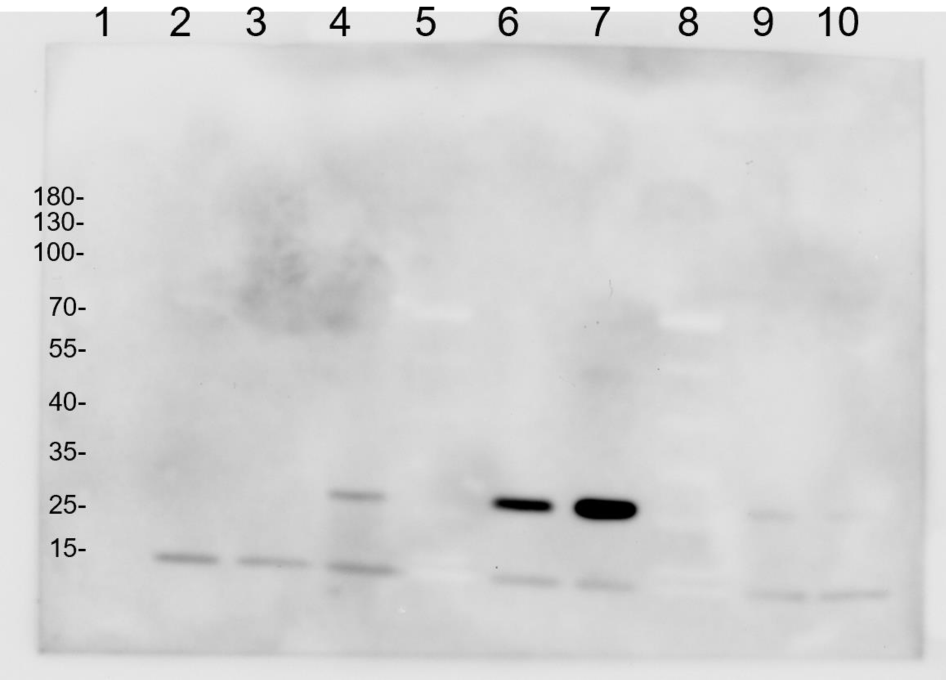

Supplementary Figure S2. Uncropped western blot picture of Figure 3. Expression of CTB-FLAG in whole cell lysate (WCL) Lanes 1: empty, 2: WCL Ty21a 3: WCL BLS-A0_B0, 4: WCL BLS-ActxB_B0, 5:

PageRuler Prestained Protein ladder (Thermo Fisher), 6-10: unrelated experiments

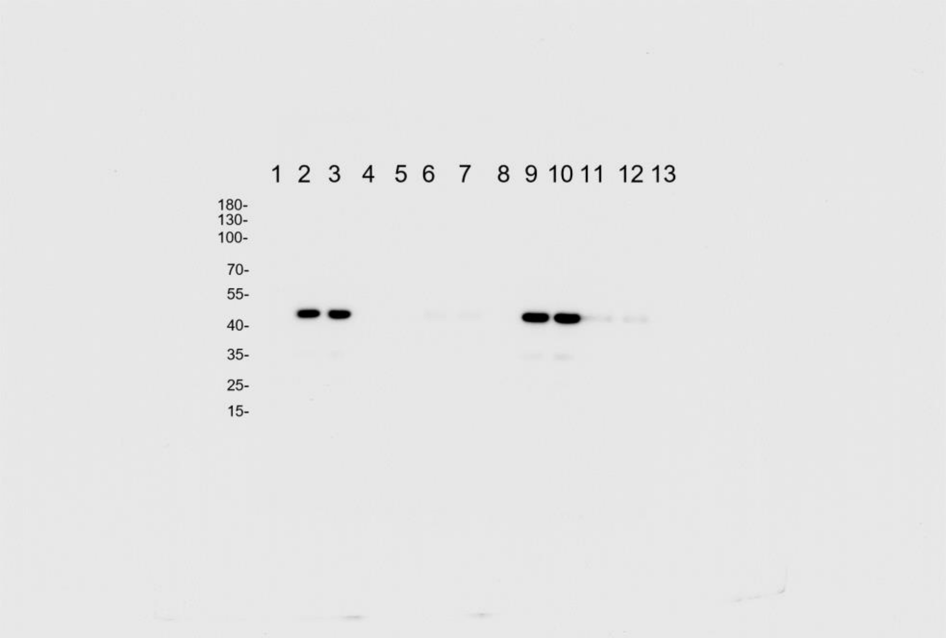

Supplementary Figure S3. Uncropped western blot picture of Figure 3. Detection of TyrS-His in whole cell lysate (WCL) and supernatant fractions (SN) Lanes 1: WCL Ty21a 2: WCL BLS-A0_B0, 3: WCL BLS-ActxB_B0, 4: PageRuler Prestained Protein ladder (Thermo Fisher), 5: SN Ty21a, 6: SN BLS-A0_B0, 7: SN BLS-ActxB_B0, 8: PageRuler Prestained Protein ladder (Thermo Fisher), 9-13: unrelated experiments.

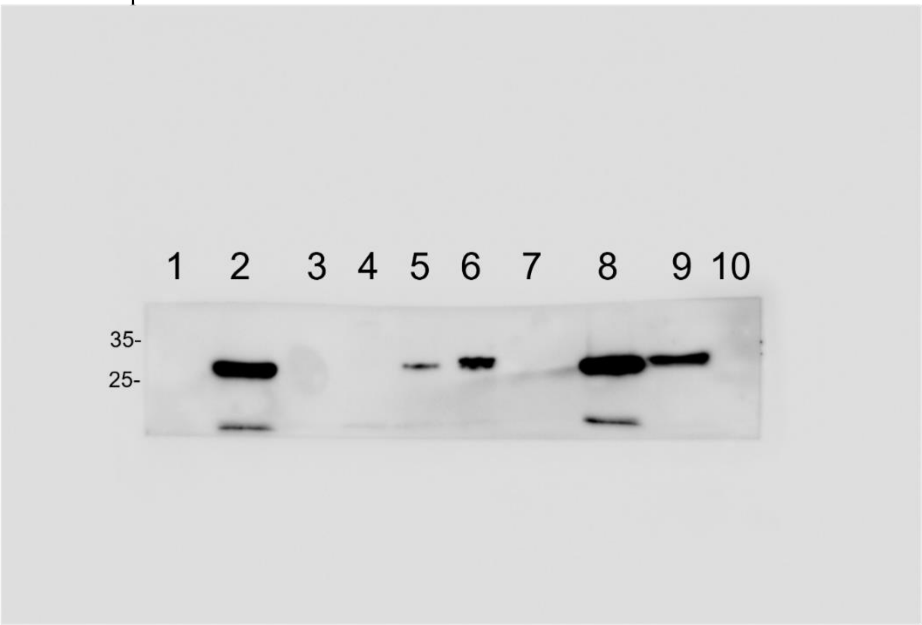

Supplementary Figure S4. Uncropped western blot picture of Figure 6. Lanes 1: Supernatant (SN) BLS-A0_B0, 2: SN BLS-ActxB_BDR, 3: PageRuler Prestained Protein ladder (Thermo Fisher), 4: Whole cell lysate (WCL) BLS-A0_B0, 5: WCL BLS-ActxB_BDR, 6: WCL BLS-ActxB_BDR +0.2% arabinose, 7: SN BLS-A0_B0, 8: SN BLS-ActxB_BDR, 9: SN BLS-ActxB_BDR +0.2% arabinose, 10: PageRuler Prestained Protein ladder.

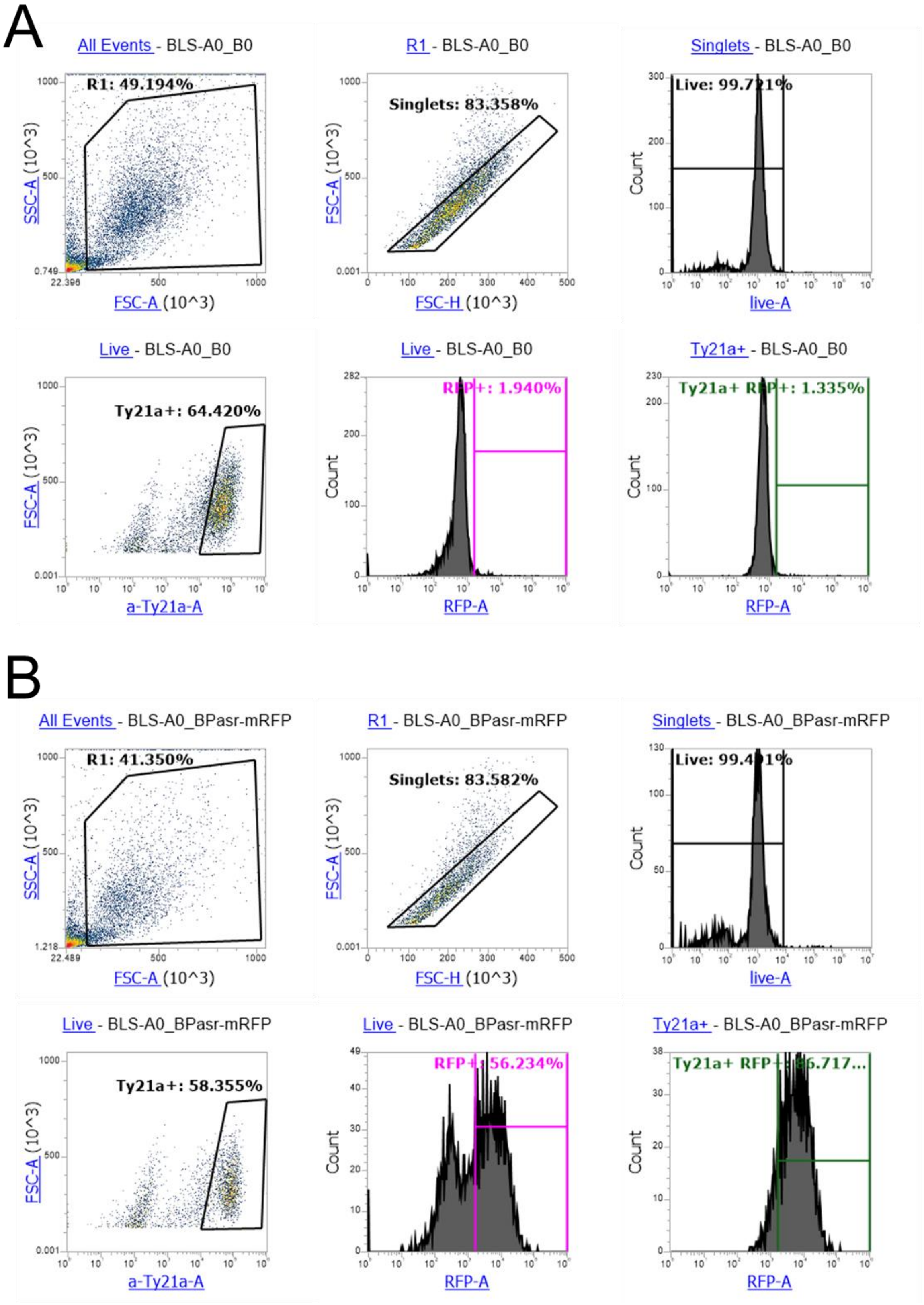

Supplementary Figure S5. Gating strategy for flow cytometry experiments. Cells were gated against debris (gate “R1”), for singlets (“Singlets”), live cells (“Live”) and subsequently for signal of anti-Ty21a staining (“Ty21a+”) or RFP (“RFP+”). The Ty21a+ population was further subgated for RFP signal (“Ty21a+RFP+”). Gates were set based on non-infected (Ty21a+ gate), or BLS-A0_B0 infected cells (RFP+

gate, see panel (A)). Panel (B) shows infection with BLS-A0_BP$_{asr}$-mRFP. The data was analyzed with the Attune™ Cytometric Software v5.2.0.

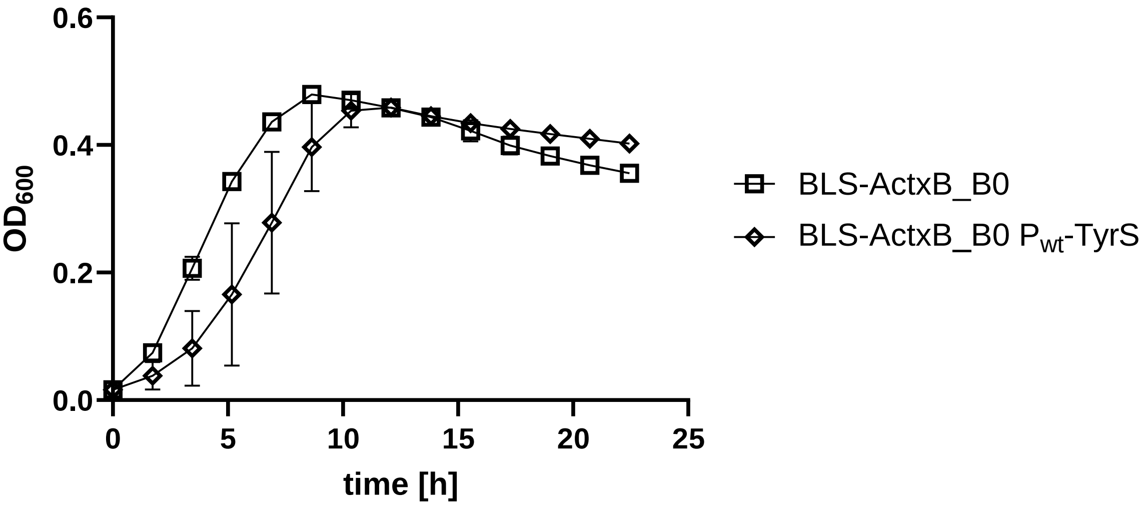

Supplementary Figure S6. Growth curve analysis of BLS-ActxB_B0 Pwt-TyrS.

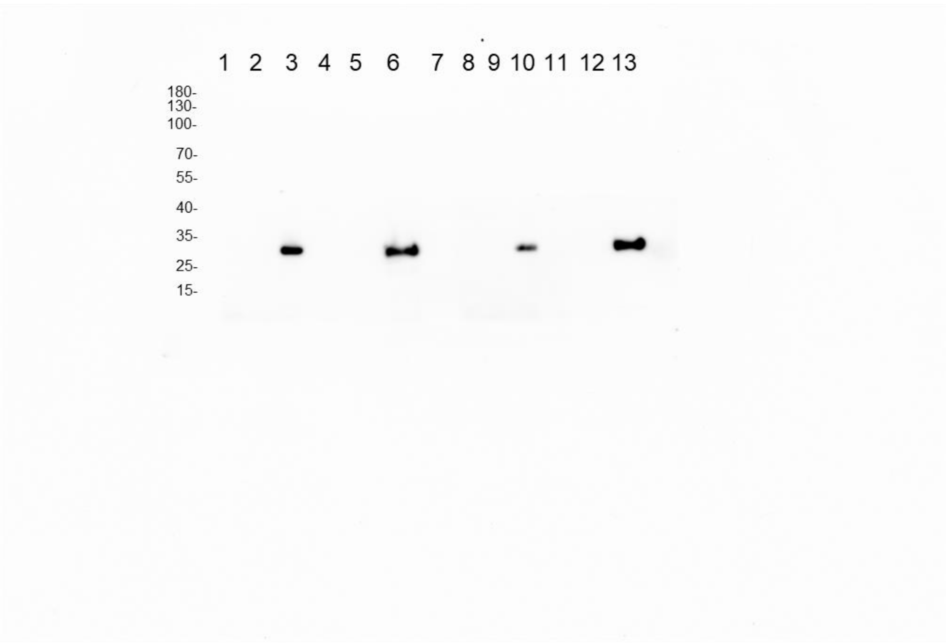

Supplementary Figure S7. Uncropped western blot picture of Figure 3F. Expression of CTB-FLAG in whole cell lysate (WCL) and TCA precipitated supernatants (SN) Lanes 1: WCL Ty21a, 2: WCL BLS-A0_B0, 3: WCL BLS-ActxB_B0, 4: SN Ty21a, 5: SN BLS-A0_B0, 6: SN BLS-ActxB_B0, 7: PageRuler Prestained Protein ladder (Thermo Fisher), 8: WCL Ty21a, 9: WCL BLS-A0_B0, 10: WCL BLS-ActxB_B0, 11: SN Ty21a, 12: SN BLS-A0_B0, 13: SN BLS-ActxB_B0.

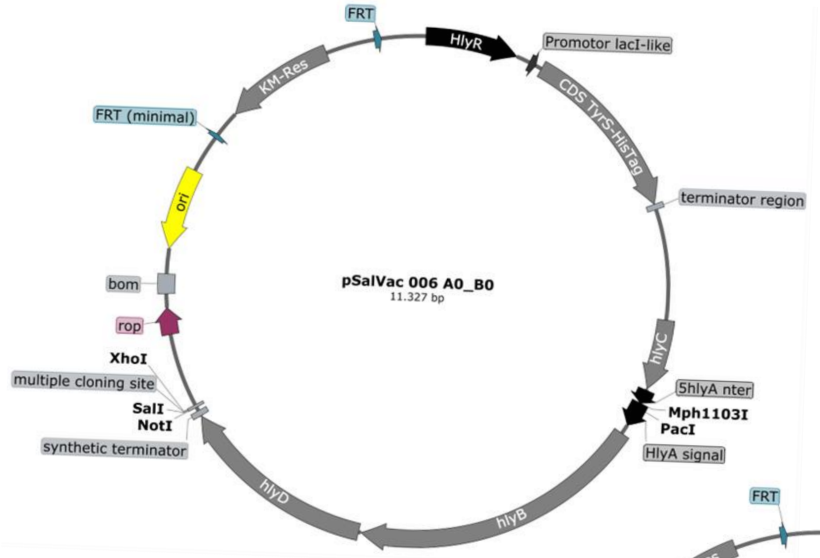
pSalVac 006 A0_B0
11.327 bp
FRT
HlyR
Promotor lacI-like
CDS TyrS-HisTag
terminator region
hlyC
5hlyA nter
Mph1103I
PacI
HlyA signal
hlyB
hlyD
synthetic terminator
NotI
SalI
multiple cloning site
XhoI
rop
bom
ori
FRT (minimal)
KM-Res

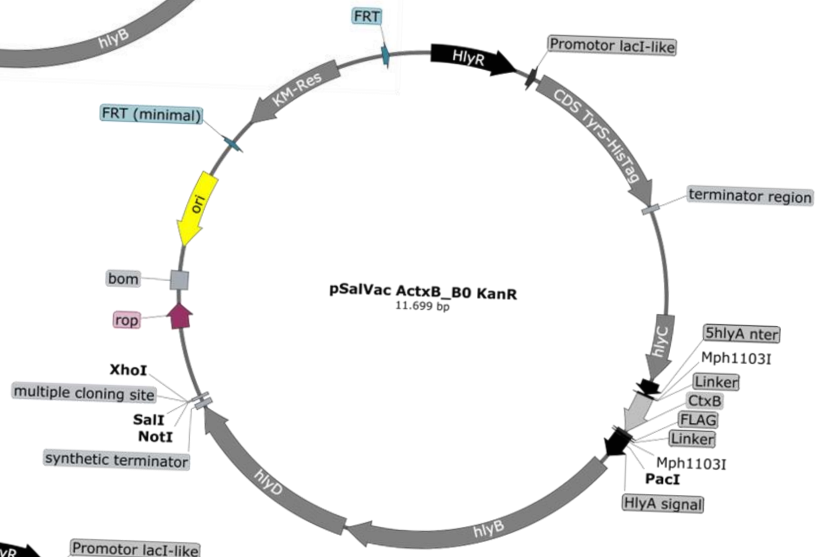
pSalVac ActxB_B0 KanR
11.699 bp
FRT
HlyR
Promotor lacI-like
CDS TyrS-HisTag
terminator region
hlyC
5hlyA nter
Mph1103I
Linker
CtxB
FLAG
Linker
Mph1103I
PacI
HlyA signal
hlyB
hlyD
synthetic terminator
NotI
SalI
multiple cloning site
XhoI
rop
bom
ori
FRT (minimal)
KM-Res

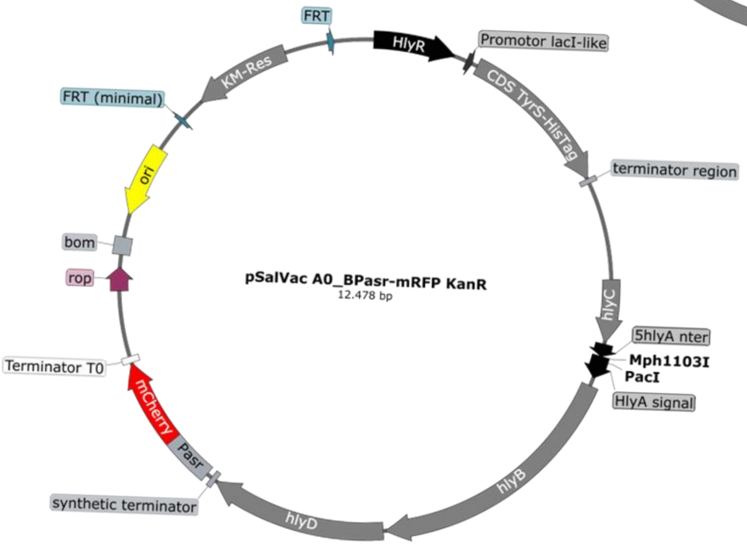
pSalVac A0_BPasr-mRFP KanR
12.478 bp
FRT
HlyR
Promotor lacI-like
CDS TyrS-HisTag
terminator region
hlyC
5hlyA nter
Mph1103I
PacI
HlyA signal
hlyB
hlyD
synthetic terminator
Pasr
mCherry
Terminator T0
rop
bom
ori
FRT (minimal)
KM-Res

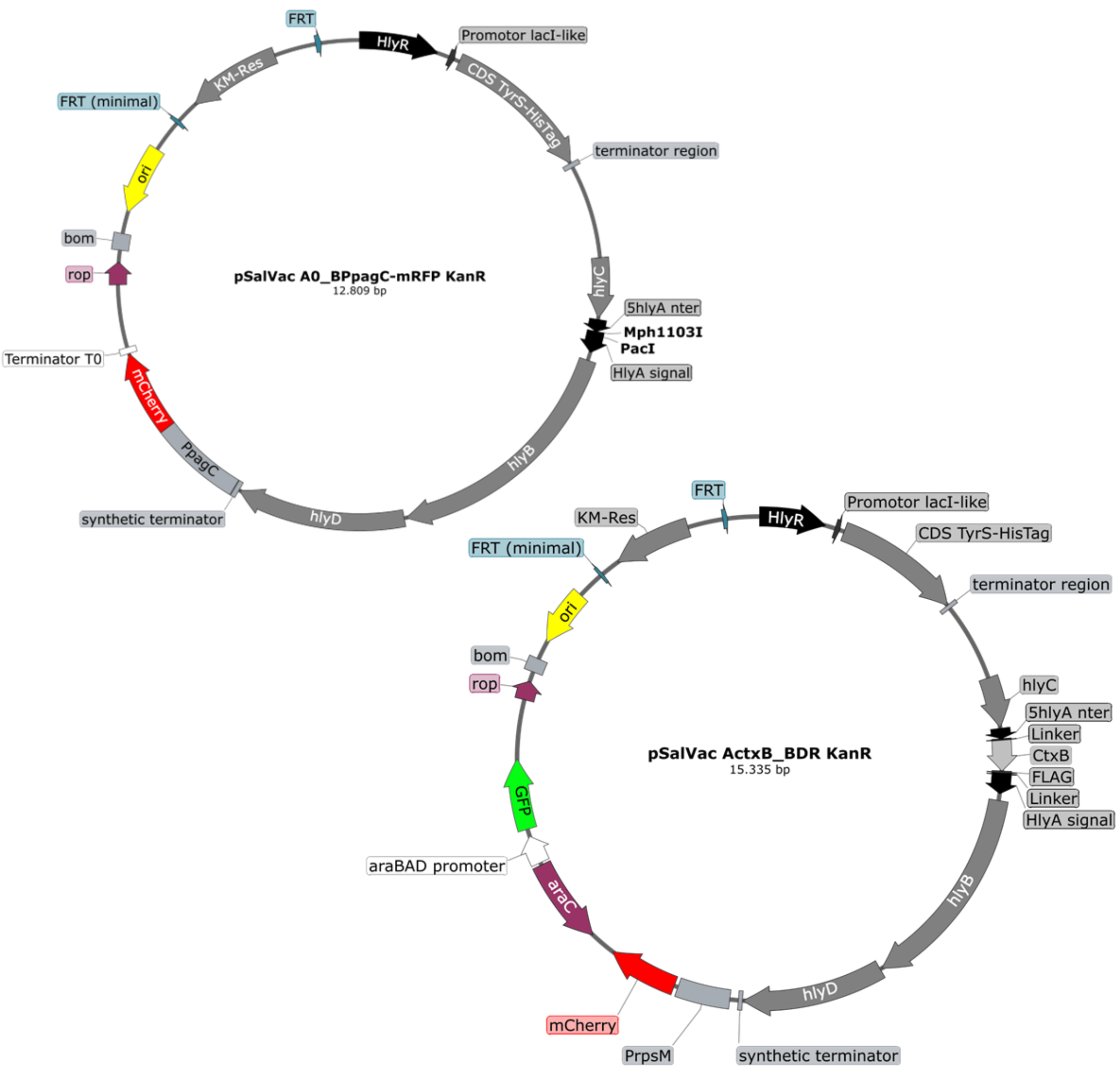

Supplementary Figure S8. Plasmid maps of key vectors used in this study.

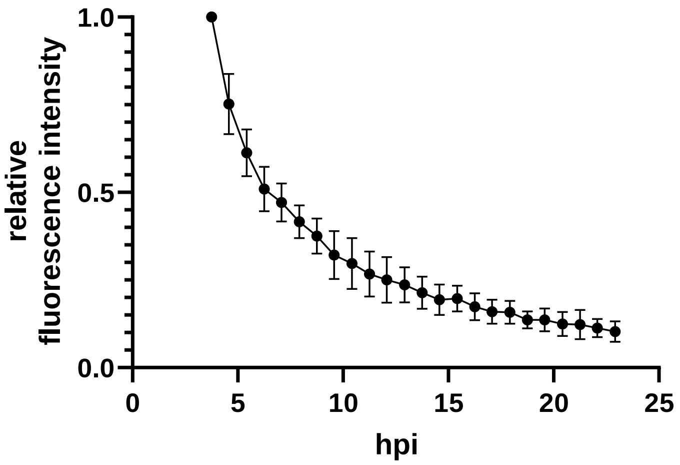

Supplementary Figure S9. Relative mRFP fluorescence intensity during live cell imaging infection with BLS-ActxB_B0 in hMDM. The fluorescence intensity of mRFP measured via live cell infection

microscopy was normalized to the first measured frame at 3.75 h past infection (hpi). Three independent experiments were performed in hMDMs differentiated from PBMCs from two different donors.
