# Supplementary material for: The JMU-SalVac-System: A Novel, Versatile Approach to Oral Live Vaccine Development
Source: Vaccines (Basel). 2024 Jun 20;12(6):687. doi: 10.3390/vaccines12060687 (PMC11209359; doi:10.3390/vaccines12060687)
Supplement: Supplementary file 1 [file vaccines-12-00687-s001.zip › Supplementary Tables.pdf]

Table S1. Primers.

**Primer**		
Mut-pKD3-SpeI-forward	GTG ATC TTC CGT CAC TAG TAG GCG CGC CGA AG	BcuI-site
Mut-pKD3-SpeI-reverse	CTT CGG CGC GCC TAC TAG TGA CGG AAG ATC AC	BcuI-site
SpeI-tyrS-EPK-forward	AAA AAA ACT AGT GTT CCC TGT ATA AAA ACC AGG GGG	BcuI-site
tyrS-EPK-SpeI-reverse	TTT TTT ACT AGT GTA AAT TCC TGG AGC TGA AGC AGA AG	BcuI-site
Ter-HisTag-1-forward	CCC CCT TTC CAC TTA ATG CAT TAG TGA TGG TGA TGG TGA TGT TTC CAG CAA ATC AGA CAG TAA TTC	x6HisTag
SpeI-Ter-HisTag-2-forward	AAA AAA ACT AGT GTT CCC TGT ATA AAA ACC AGG GGG AGT GAT TTC TCA CTC CCC CTT TCC ACT TAA TGC ATT AG	BcuI-site
tyrS-HisTag-reverse	CAT CAC CAT CAC CAT CAC GCA AGC AGT AAC TTG ATT AAA	x6HisTag
knockout-forward	**GTG TAC CGG CAA AGG TGC AGT CGT TTT ATA CAT GGA GAT TTT GAT GGC A**GT GTA GGC TGG AGC TGC TTC	
knockout-reverse	**GAT AGT GAC AGC GTT GGA GGC GAT AGT CTT ACG CGC CTG ACC ACG TGA CGG** ATG GG A ATT AGC CAT GGT CC	
SpeI-IS2-Deletion-forward	AAA AAC TAG TGA TAA TGG TTC ATG CTA CCG GGC GAA TG	BcuI-site
IS2-Deletion-BamHI-reverse	GTT TTG GGA TCC ACC CTG ATG GCT CTG	BamHI-site
LacI-Prom.for	AAA AGT CGA CTA GTG CTA GCG ACA CCA TCG AAT GGC GCA AAC CTT TCG CGG TAT GGC AT**G** ATA GCG CCC G**AA GTC GTG TAC CGG CAA AGG TGA GTC G**	SalI/SpeI-sites
LacI-Ter-rev	AAA AAA GTC GAC TAG TGG TAC CAA AAT GCC GCC AGC CCA AAC	SalI/SpeI-sites

	TGG CGG CCG TGG ATT A**GT GAT GGT GAT GGT GAT G**TT TCC AGC	
pMO-tyrS-screen-forward	CCC TGA ATC TCC AGA CAA CCA ATA TCA	screening
pMO-tyrS-screen-forward	CCC GTA CAA ATT CTA CCA GTT CTG GA	screening
CH19 bsite mcs rev	TCGACGCGGCCGCATGATTCAAATGGATCAACATCTCGAGGAGCTCG	Insertion of MCS in pSalVac 001 A0_B0
CH18 bsite mcs fwd	TCGACGAGCTCCTCGAGATGTTGATCCATTTGAATCATGCGGCCGCG	Insertion of MCS in pSalVac 001 A0_B0
CH56 IF TyrS fwd	CACCAGCGTGTTCGTGTCATGTGCCACCTGACGTCTAAGAA	Excision of AmpR promoter
CH58 IF Kmres rev	ATGACACGAACACGCTGGTGCGTGTCGCCCTTATTCCCTT	Excision of AmpR promoter
CH61 B1006 rev	GGCCGCaaaaaaaaaccccgccctgtcaggggcggggttttttttGAGCT	Synthetic terminator
CH60 B1006 fwd	CaaaaaaaaaccccgcccctgacagggcggggttttttttGC	Synthetic terminator
CH110 IF4 gen6 Km fwd	GCGGCCGCgtcgacCTCGAGgaacatatccatcgcgtccg	Deletion of non coding elements
CH235 ctxB flag	tcgtcgtcgtctttgtagtcgttcgccatggagatcgccgc	Insertion of ctxB in A-site
CH239 flp KO	ATGACACGAACACGCTGGTGaagatcccccacgctg	Deletion of downstream KanR FRT site
CH53 IF 204 fwd	acaaagcacgaaagATGCATCAGAAGCGGC	Insertion of ctxB in A-site
CH146 pasr fwd	CCATGGTCCCTCAAGCGTTAaataatccggcgatagcaacg	Insertion of Pasr in B-site
CH151 asr rev	tttgttacccccgatttggttttc	Insertion of Pasr in B-site
CH156 mCherry fwd	accaaatcgggggtaacaaaatggtgagcaagggcgag	Fusion of Pasr to mRFP in B-site

CH155 mCherry rev	agaccgagcgttctgaacaactacttgtacagctc gtccatgc	Fusion of Pasr/PpagC to mRFP in B-site
CH157 PpagC fwd	CCATGGTCCCTCAAGCGTTAgttaac cactcttaataataatgggttttatagc	Insertion of Pasr in B-site
CH159 PpagC rev mod	TTTGTTACCCCCGATCTACTTATTA tttacggtgtgtttaaacacc	Insertion of Pasr in B-site
CH161 mCherry PpagC	AGTAGATCGGGGGTAACAAAatggt gagcaagggcgag	Fusion of PpagC to mRFP in B-site
CH108 dualrep fwd	gctgaaGCgtcgacCTCGAGGGGATCC CGGAGTTCATGC	Insertion of dual reporter cassette
CH109 dualrep rev	cggacgcgatggatatgttCcGCCATAGTG ACTGGCGATG	Insertion of dual reporter cassette

Table S2. Raw data for Figure 3.

Strain	Day 0	Day 1	Day 2	Day 3	Day 4	Day 5	Day 6
Ty21a-ActxB_B0 Kan	92/102 (90.2 %)	79/102 (77.5%)	25/102 (24.5%)	13/102 (12.7%)	7/102 (6.9%)	2/102 (2.0%)	0/102 (0%)
BLS-ActxB_B0 KanR	102/102 (100.0%)	102/102 (100.0%)	102/102 (100.0%)	102/102 (100.0%)	102/102 (100.0%)	102/102 (100.0%)	102/102 (100.0%)
Ty21a pMKhly-ctxB	88/102 (86.3%)	73/102 (71.6%)	6/102 (5.9%)	7/101 (6.9%)	3/102 (2.9%)	2/102 (2.0%)	3/102 (2.9%)
BLS-ActxB_B0	102/102 (100.0%)	n.d.	n.d.	n.d.	n.d.	n.d.	102/102 (100.0%)
