# Supplementary figures and images for: The JMU-SalVac-System: A Novel, Versatile Approach to Oral Live Vaccine Development
Source: Vaccines (Basel). 2024 Jun 20;12(6):687. doi: 10.3390/vaccines12060687 (PMC11209359; doi:10.3390/vaccines12060687)

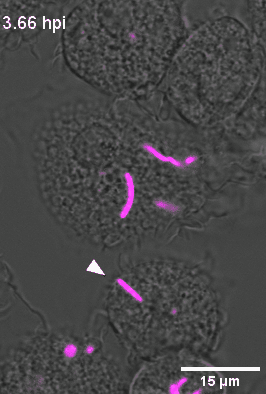

Supplement: Supplementary file 1 [file vaccines-12-00687-s001.zip › Supplementary Movie S1.gif]
